# Healthcare teams' ethical obligations towards care of adolescents' living with human immunodeficiency virus disease

**DOI:** 10.1002/nop2.1728

**Published:** 2023-04-05

**Authors:** Renatha S. Joseph, Gasto Frumence, Nathanael Sirili, Connie M. Ulrich

**Affiliations:** ^1^ Department of Bioethics and Health Professionalism, School of Public Health and Social Sciences Muhimbili University of Health and Allied Sciences Dar es Salaam Tanzania; ^2^ Department of Development Studies, School of Public Health and Social Sciences Muhimbili University of Health and Allied Sciences Dar es Salaam Tanzania; ^3^ Biobehavioral Department, School of Nursing, Department of Medical Ethics and Health Policy, and New Courtland Center for Transitions and Health University of Pennsylvania Philadelphia Pennsylvania USA

**Keywords:** adherence, adolescents, ethical obligation, healthcare providers, HIV care and treatment

## Abstract

**Aim:**

To explore healthcare team members' perceptions of their ethical obligations toward HIV‐positive adolescents and their enrolment in and adherence to antiretroviral therapy among adolescents attending a Care and Treatment Center (CTC) in Temeke Regional Referral Hospital in Tanzania.

**Design:**

This is a descriptive exploratory qualitative study.

**Methods:**

A total of 16 healthcare team members were purposively selected from the hospital CTC to participate in in‐depth qualitative interviews. With the aid of NVivo software, qualitative thematic analysis was used to analyze the information.

**Results:**

Five themes on ethical obligations emerged: (1) informing adolescents of their status before enrolment to the HIV CTC, (2) securing adolescents’ confidential information, (3) disclosing adolescents' HIV status, (4) informing others about the adolescent’s HIV status; and (5) offering reproductive health education for adolescents living with HIV.

**Conclusion:**

The healthcare team faces many ethical challenges in the care and support of adolescents who enroll in an HIV CTC in Tanzania. Differing ethical obligations must be balanced with the needs of adolescents and their parents in discerning what is in the best interest of the adolescent and advocating for life‐saving treatment.

## 1 INTRODUCTION

Adolescents living with human immunodeficiency virus (HIV) in Tanzania are chronically ill and in need of healthcare services to maintain their health and well‐being; yet, many may not receive life‐saving antiretroviral therapy (ART). The World Health Organization (WHO) has argued for fair and equitable distribution of ART to vulnerable populations while recognizing the many barriers to treatment (2004). Physicians, nurses and other healthcare team members who work with vulnerable populations in low‐ to middle‐income countries have an ethical obligation to provide quality care to their patients, including the promotion of health and the avoidance of harm (Medical Council of Tanganyika, 2005; Tanzania Nurses and Midwives Council, 2007). Caring for HIV‐positive adolescents in Tanzania presents ethical challenges for all stakeholders, including the healthcare team. Both professional and ethical obligations arise to educate patients on their diagnosis, prognosis and other issues that might be of concern in their day‐to‐day lives. This also includes obtaining parental informed consent and paediatric assent in adolescent care and treatment for HIV and other tests or procedures (Katz & Webb, 2016).

The primary obligation of the healthcare team is to respect and promote the welfare and dignity of the client; however, in adolescent care, it is challenging because parents also share a unique role in determining what is best for their children. Indeed, adolescents' dependence on others, such as their parents or designated primary caregivers, can create a sense of vulnerability and power imbalances in the relationship (Damuliraa et al., 2019). Children have the right to participate in decisions that affect them and the right to be heard, with their privacy and confidentiality respected by the healthcare team (MacPherson, 1989; WHO, 2021). At the same time, parents or primary caregivers have the right to know their adolescent's problems and how the treatment care plan can properly support the adolescent (UNICEF, 2021). There are times, however, when there is no agreement on what should be done for the adolescent, and ethical dilemmas arise for the healthcare team in discharging their duty of respecting both parental and adolescents' rights (Kilkelly & Donnelly, 2006; Guadarrama‐orozco, 2015).

In some families, a child's HIV status is known from infancy; yet the family does not disclose this information to the growing child and provides HIV medication to the child by calling it vitamins or something similar. In adolescent HIV care and treatment, ethical issues often encountered by the healthcare team can include the involvement of adolescents in the decision for HIV testing and issues surrounding disclosure of an adolescent's HIV status to the adolescent, such as privacy concerns and the potential for stigma and discrimination that the adolescent may suffer (Etemad, 2018; Ulrich et al., 2010). The tension between the duty to treat and the duty to inform a patient who does not know their HIV status is ever present in the relationship between the adolescent, the parent or primary caregiver and the healthcare team. Informed consent is a central tenet of the patient–provider relationship, yet adolescents less than 16 years of age need parental consent, according to national guidelines for HIV testing and care enrolment in Tanzania (National AIDS Control Program, 2015). Parental consent for minors is the requirement by Tanzanian law, although this can also raise ethical concerns on disclosure risks and balancing what is ethical and what is legal in the context of health delivery in Tanzania.

Literature on healthcare teams' perceptions of the ethical issues in adolescents' HIV care and treatment in Sub‐Saharan Africa, especially in Tanzania, is very scarce. Therefore, this study aimed to explore healthcare team members' perceptions of their ethical obligations to HIV‐positive adolescents and their enrolment in and adherence to ART among adolescents attending a Care and Treatment Centre (CTC) at Temeke Regional Referral Hospital in Tanzania.

## 2 METHODOLOGY

An exploratory qualitative descriptive design was used to explore healthcare team members' ethical obligations to adolescents' enrolment in and adherence to ART in Temeke Regional Referral Hospital, Tanzania. This design was appropriate because little is known about the healthcare team and how they interact with HIV‐positive adolescents who attend a CTC in Tanzania and any challenges they may experience in their roles. This study was part of a larger study on the ethical issues of HIV care and treatment for adolescents in Tanzania (Joseph et al., 2022).

### 2.1 Participant recruitment and data collection

Prior to recruitment of participants, institutional review board approval was received from Muhimbili University and the National Research Ethics Review Committee in Tanzania. A total of 16 healthcare team members at the CTC were recruited to the study. The CTC is a public facility that is designed to enrol people living with HIV and to provide them with HIV treatment and care to prolong their lives (Kilama, 2015). The Temeke CTC usually sees more than 700 adolescents per month who are diagnosed with HIV, and the healthcare team consists of 40 members. These members include physicians, nurses, social workers and others who are dedicated to working with young people and their families.

The principal investigator (PI) (RSJ) enrolled participants with the assistance of two trained research assistants with a background in Bioethics. Participants were purposively selected because they specifically worked with adolescents living with HIV who were attending the CTC at Temeke Regional Referral Hospital and who were integral to providing care for adolescents at this centre. Written informed consent was obtained from all participants.

The semi‐structured interviews were conducted in Swahili—the natural language of the participants—and also in private rooms at Temeke CTC during working hours. The average duration of each interview was 45 minutes. The interview guide included open‐ended questions asking participants to describe their perceived obligations in enrolling and caring for CTC youth in Temeke. For example, participants were asked about their decision‐making process and what guides these processes in adolescents' HIV care and treatment. Participants were also asked to provide their thoughts on what might influence adolescents to attend the clinic and what might help them to take their medications as required. Saturation was achieved with 16 qualitative interviews (Whittemore et al., 2001). The study was conducted from January to August 2020.

### 2.2 Data analysis

Descriptive statistics were used to analyse the participants' demographic characteristics, including age, gender, working duration and their occupation or professional duty at the CTC. Data were transcribed verbatim by the PI, and each transcript was uploaded to NVivo 12 software, which assisted in data coding. Open coding was also conducted on printed hardcopies before uploading them to the software. A process to support the rigour of the qualitative data was followed, as outlined by qualitative researchers (Braun & Clarke, 2006) and also reported elsewhere (Joseph et al., 2022). This process includes a series of steps that help to identify themes within the data. For the first step, the PI and a research team member (GF and NS) reviewed the transcripts to become familiar with the data and to assess any outstanding concerns. Initial codes were then generated to organize the data in a meaningful way by reviewing the phrases, sentences and paragraphs expressed by participants. Open coding was used to develop initial codes, and codes were modified by examining related codes and refining as needed as the coding process continued. Some of the codes were collapsed into initial themes for review and consideration by the research team. The codes were then organized into broader themes that seemed to specifically describe the healthcare team's sense of obligation in adolescents' HIV care and treatment. Themes were then reviewed to identify whether they were supported by the data. Related themes were combined to form a single theme representing participants' responses, followed by interpretation of the relationships among the themes.

Trustworthiness of the qualitative data (i.e., do the themes identified by the coding and analysis fully reflect the views of the interviewees) is critical to ensuring the accuracy of the findings. First, the first author (RSJ) spent some time in the field and was familiar with the local environment, which added credibility. Second, the different perspectives of members of the research team, who had varying levels of familiarity with the setting, were incorporated. Research team members have expertise in bioethics, public health, paediatric medicine, social sciences and health system management. Third, transferability, a determination of the applicability of the results to other contexts and a method of assessing the reliability of the data, was assessed (Glaser, 2004; Lincoln & Guba, 1985). Here, transcripts were translated into English, and emerging issues were discussed among members of the research team, enriching the interpretation of the data by balancing perspectives representing diverse backgrounds and qualitative expertise. The PI also recognized and reflected on her position as study leader and how her background in paediatric medicine and as an African researcher may affect the overall research process (Holmes, 2020). These measures add credibility to the presentation of the participants' opinions. Finally, our research also follows the Consolidated Criteria for Reporting Qualitative Research (COREQ) that recognizes and addresses the importance of research teams, study design, sample size and analysis and results (Tong et al., 2007).

## 3 RESULTS

### 3.1 Demographic characteristics of participants

Of the 16 healthcare team members interviewed, most were female, with a female‐to‐male ratio of 2:1. This included two physicians, two clinical officers, two peer leaders, eight nurses, one data clerk and one pharmacist. Ages ranged from 20 to 55 years (mean = 36.3 years), and their work experience in terms of years worked at the Temeke CTC ranged from 2 to 10 years (mean = 5 years).

### 3.2 Qualitative themes

Five qualitative themes emerged from the data. These themes included specific professional and ethical duties, such as duties to inform adolescents of their disease before HIV testing and enrolment in HIV care and treatment. Other responsibilities were to protect confidential information of young people, inform young people of their HIV status and the challenges they face in disclosing their HIV status and provide reproductive health education to young people living with HIV to reduce the prevalence of HIV and prevent infection—and finally, to help prevent the spread of HIV and ultimately inform others who may be at risk of HIV infection from their partners.

### 3.3 Theme I: Obligations to inform adolescents before HIV testing and enrolment in HIV care and treatment

Participants reported that they have obligations to inform adolescents about HIV testing and the possibilities of living with HIV. Counselling was also an important professional and ethical obligation reported by peer leaders, nurses and physicians to support newly diagnosed adolescents. For example, as one participant shared, ‘We counsel adolescents before going for an HIV test that they may test either negative or positive. We then do posttest counselling depending on the test results. Those that are living with HIV are then enrolled to the care and treatment of the HIV clinic’ (Nurse counselor, HCP #15). One participant noted that ‘there is a guideline for helping adolescents who are living with HIV and also for those who tested negative for HIV’, recognizing that support is also needed for those who are not HIV positive. Another participant reported on the important role of informed consent as a continuing process for adolescents and described it in the following way:

The adolescent may be advised by a doctor to be HIV tested through Provider Initiated Testing and Counseling (PITC), the program which tries to test and enroll many people living with HIV when they come to [the] hospital for other problems. The doctor will refer an individual to [a] counselor; therefore, as counselors we need to guide [the] adolescent again before testing, we tell him/her the possibilities and affirm his/her willingness to be tested for HIV. Depending on [the adolescent's] age, we may test without their parents' consent from 16 years [of age]. (Nurse counselor, HCP #04)

The study participants also revealed that there are several other ways to have adolescents receive information about HIV testing, including mass campaigns, sexual contact tracing and group counselling, such as in football clubs as mentioned here:

Adolescents who are in the CTC program are advised by peer leaders to have their sexual partners tested. Street campaigns, sports/football groups, these activities are done in different places to encourage adolescent HIV testing and enrollment in care. (Expert patient, HCP #01)

### 3.4 Theme 2: Ethical obligations to secure adolescents' confidential information

Issues related to confidentiality were highlighted by several study participants, especially because parents have legal rights to their children's information for those who are under age 18. This tension was noted by one of the participants in this way: ‘We keep information secret between the counselor and the adolescent, even from the parent, until the adolescent is ready for disclosure to their parent or caretaker’. (Nurse counselor, HCP #03).

Participants reported the ethical dilemmas raised by keeping the adolescent's confidential information from parents while HIV guidelines in Tanzania allow for testing from ages 15 to 18. However, adolescents need parental support, as this participant said, ‘We have to disclose adolescents' information to parents or [their] family for smooth care’. (Clinical officer, HCP #11).

### 3.5 Theme 3: Disclosure obligations about adolescents' HIV status

As noted above, the family may not disclose to the growing child his or her HIV status, providing medication as vitamins or similar. Participants perceived that the healthcare team members have professional and ethical obligations to make sure adolescents living with HIV take their required medication appropriately, because it affects their long‐term health outcomes. Full disclosure is of paramount importance for adolescents so they can understand the medication they are using and take it according to directions. Thus, there was a feeling that the healthcare team needed to disclose the adolescent's HIV status to the adolescent, especially when it was not disclosed by the family, as noted by the two quotes below:

First, information is supposed to be disclosed by family members and not service providers. Depending on family understanding, most of the adolescents are taken care of by their grandmothers. Grandmothers see this information as too heavy for her grandchild. So, education to the grandmother should be continuous. (Nurse counselor, HCP #02)

All adolescents should know their status by 7 years, but some parents/caregivers say my child is still young, just wait first, but we make sure [that by] up to 10 years [of age] all of them know their status and know why they are using medicine. And, what is the importance of this medicine, so we start to train them early. (HCP #03)

Participants also reported that non‐disclosure of adolescent HIV status can have detrimental effects on adolescents' adherence to medication and ultimately their health. Some of the adolescents stop taking medication because for many years their parents did not tell them why they were taking it. As noted by one participant:

There is one adolescent here whom we gave [HIV] information, and he said I don't want to use medicine [because] my mother didn't tell me about [my] HIV status, but you [now] tell me this information. It took a long time, but later on we came to [an] agreement. (Nurse counselor, HCP #03)

### 3.6 Theme 4: Challenges healthcare teams face during disclosure of HIV status to the adolescent

Participants reported the ethical challenges they face when parents or primary caregivers fail to disclose the adolescent's HIV status. Adolescents on ART, for example, will keep asking why they are taking medicine daily. Some parents lie to their children telling them that they have sickle cell anaemia, instead of telling them the truth about their HIV status. So, when a healthcare team member wants to disclose the information, it is difficult to tell them that it is not sickle cell anaemia but HIV. This is illustrated by one of our participants saying, ‘Sometimes adolescents are told to have other problems like sickle cell anemia, so you see, you need to differentiate what sickle cell is from HIV, give the right information, something that the adolescent doesn't understand, and it consumes time. So that's why it needs to be done in phases’. (Nurse counselor, HCP #02).

Participants reported that frank discussions are needed with adolescents. One participant said that this helps to reduce challenges that the healthcare team experiences: ‘To reduce challenges, frank disclosure should be done as early as seven years of age, which starts with encouraging parents/caregivers to start disclosure [discussions]’. (Nurse counselor, HCP # 04)

During disclosure sessions, continuation with the same group of providers is also important for clarity and understanding of information being disclosed to the adolescent. Sometimes, however, the CTC service lacks the component of continuity of care because of differing physicians, nurses and counselors as seen in the quotes below:

So, the problem is when he/she comes for the next phase, is he/she going to meet with you or not? Adolescents sometimes gets confusing information from home and different care providers [at the CTC]. (Nurse counselor, HCP #02)

Files are there but, in the files, you fill that he/she has come [to the CTC] and I gave medicine. I started at stage one, then I proceeded. But there it has no details on what happened previously, and everyone has their own understanding on how to work. I as a counselor and other counselors there, [find that there] are things that we do differently during [an] adolescent counselling session. (Nurse counselor, HCP #02)

Participants also reported on their fear of hurting a child as one reason for the healthcare team to hesitate on informing adolescents about their HIV status. This was stated eloquently by one of the participants.

Surely adolescents get hurt so much when they find themselves living with HIV, also when you tell them they are going to use the medications every day for the rest of their lives they feel like it's not fair. (Nurse counselor, HCP #15)

Participants reported that despite all the challenges, the healthcare team must find a way to make sure disclosure takes place, as noted by one of the participants: ‘Most times we do use the people who are closely related to the children, because we normally meet these children daily and every child has a person s/he understands and are most depending on the relationship’. (Psychiatrist, HCP #14)

### 3.7 Theme 5: Offering reproductive health education for adolescents living with HIV

Reproductive health education was an important theme, whereby the team perceived this type of knowledge was protective for adolescents living with HIV and to help them make informed decisions with their partners and to avoid infecting others. As one participant said: ‘I work with adolescents living with HIV, so I talk to them about partner protection and testing in case there is the possibility of being infected (we said it in index contact tracing)’. (Nurse counselor, HCP #02)

Other participants spoke to the need to start early counselling about sexual relationships, especially when they may lack family support and suffer from economic hardships. The two quotes below address these concerns:

Counselling about sexual relationship starts at puberty and is done on Saturday when no one else is around except adolescents because we talk the same language for easy understanding. (Nurse counselor, HCP #03)

In my experience, there are adolescent girls whom we teach but suddenly they come back pregnant, this is because of economic hardship and lack of proper families that take care of adolescents living with HIV. We have obligations to make them understand the importance of abstaining or use of a condom. (Medical doctor, HCP #05)

## 4 DISCUSSION

Healthcare teams have professional and ethical obligations towards their adolescent patients. In this study, obligations of the healthcare team included a variety of issues: confidentiality of patient information, disclosure of HIV status for those who were tested at a young age and prevention of HIV infection of others through disclosure to sex partners and reproductive health education to adolescents living with HIV.

In this study, informing adolescents and getting their consent for adolescent treatment and care was perceived as a way to improve adolescents' adherence to ART and HIV care in general, but obtaining informed consent of adolescents presents many challenges, including an adolescent's capacity to understand and make important life‐altering decisions. Depending on the age of the adolescent, informed consent requires the healthcare team's assessment of the adolescent's capacity for autonomous decision‐making (Michaud et al., 2015; WHO, 2021). Adolescents have the right to information, although each adolescent is different in their level of maturity and what their worries might be with an HIV diagnosis, as stipulated in the International HIV/AIDS Alliance Good Practice Guide (Armstrong et al., 2017). Despite the need for adolescents' informed consent or assent, parents have the right to decide for their children unless those minors are married, parents or sexually active, according to the Tanzanian Ministry of Health & Social Welfare (Pampati et al., 2019). The healthcare team must balance their professional and ethical obligations to do no harm to the adolescent and maintain trusting relationships when parental authority and legal guidelines dictate different decisions surrounding the care and treatment of the adolescent.

Findings from other studies confirm that the informed consent process in HIV services for adolescents raises several dilemmas, including the adolescent's insufficient capacity to make life decisions, the adolescent's economic dependency on others and the healthcare team's insufficient familiarity with laws and policies of adolescent involvement in their own care (Ho et al., 2005; Joseph et al., 2022). In this situation, healthcare teams have obligations to make sure that adolescents under their care understand the information given to them in the informed consent or assent process and the limits of confidentiality in certain cases (Pampati et al., 2019). In this study, the healthcare team at Temeke CTC reported that informed consent (or assent) has to be continuous and consistent in adolescent HIV care and treatment, but it is lacking in both. Future research should focus on the process of informed consent (and assent) and how to involve adolescents more meaningfully in this process and what this process actually means to them.

Issues of confidentiality and privacy of information remain salient concerns. Indeed, parents need to be informed about their adolescents' HIV status for them to support their children in the best way possible (American Academy of Paediatrics, 2012). However, confidentiality is important in adolescent HIV care and treatment because it helps to build a trusting relationship between the client and healthcare team (Ford et al., 2016). Thus, the healthcare team may have difficulty in deciding whether to keep adolescent confidentiality or to inform the adolescent's parents for better and consistent care and financial and emotional support.

Disclosure of HIV status is required for adolescents to take responsibility for their own health as they grow older (National Department of Health South Africa, 2016). Parents are supposed to disclose the information about their adolescents' HIV status (Mandalazi et al., 2014), but some of them are worried about their children and the barriers they may encounter because of an HIV diagnosis. In this study, the healthcare team members recognized disclosure as their professional and ethical obligation. This obligation has also been recognized by participants in similar studies (Vreeman et al., 2010). Healthcare team members faced challenges in the process of disclosing HIV status to adolescents living with HIV and receiving care at the Temeke CTC—a finding similar to those from other studies (Madiba & Diko, 2021). These challenges resulted from parents' nondisclosure or delayed disclosure of HIV status to their child or from their provision of false information, such as by telling the adolescent that he or she had a less stigmatizing disease like sickle cell anaemia rather than HIV. In general, children trust their parents more than anyone else, and they believe what they have been told. Healthcare team members reported difficulty in correcting such situations. Future research is needed to better understand the adolescent–parent–healthcare team relationship and the factors that support shared decision‐making and trust among all stakeholders.

Finally, healthcare team members have an ethical obligation to inform others in case of HIV infection, including sexual partners. Informing sexual partners reduces transmission from one person to another. Reproductive education is very important in adolescent HIV services. Well‐informed adolescents will know how to protect themselves and their loved ones as they live with their HIV diagnosis. Participants from this study also recognized their obligation to prevent harm to others.

## 5 CONCLUSION

Healthcare team members recognize their ethical obligation to provide healthcare for adolescents living with HIV that meets their expectations of being treated with respect and dignity. At the same time, there is a need to inform parents about the health status of the adolescent and their care in general. This creates tension for healthcare team members to fulfill their obligations to both adolescents and parents.

## AUTHOR CONTRIBUTION

Renatha Sillo Joseph—Proposal development, seeking ethical clearance, data collection, data analysis, article preparation and submission. Gasto Frumence—Is a main supervisor; Proposal development, data analysis, article preparation. Nathanael Sirili—Data analysis and article preparation. Connie M. Ulrich—Is a co‐supervisor; Proposal development, data analysis, article preparation.

## CONFLICT OF INTEREST STATEMENT

There is no conflict of interest.

## FUNDING INFORMATION

The study is part of PhD study sponsored by ministry of Education, Science and Technology: FB.84/370/83.

## ETHICAL APPROVAL

Ethical clearance was obtained from MUHAS IRB DA.282.298/01.C/ and National Health Research Ethics Committee NIMR/HQ/R.8a/Vol.IX/3299.

## Data Availability

Data are available with request but also included in the article as qualitative themes.
